# Bearing Fault Detection Based on Empirical Wavelet Transform and Correlated Kurtosis by Acoustic Emission

**DOI:** 10.3390/ma10060571

**Published:** 2017-05-24

**Authors:** Zheyu Gao, Jing Lin, Xiufeng Wang, Xiaoqiang Xu

**Affiliations:** 1 Shanxi Key Laboratory of Mechanical Product Quality Assurance and Diagnostics, Xi’an Jiaotong University, Xi’an 710049, China; gebecca@163.com (Z.G.); wangxiufeng@mail.xjtu.edu.cn (X.W.); xu.xiaoqiang@hotmail.com (X.X.); 2 State Key Laboratory of Manufacturing System Engineering, Xi’an Jiaotong University, Xi’an 710049, China

**Keywords:** acoustic emission, correlated kurtosis, Empirical Wavelet Transform, bearing fault detection

## Abstract

Rolling bearings are widely used in rotating equipment. Detection of bearing faults is of great importance to guarantee safe operation of mechanical systems. Acoustic emission (AE), as one of the bearing monitoring technologies, is sensitive to weak signals and performs well in detecting incipient faults. Therefore, AE is widely used in monitoring the operating status of rolling bearing. This paper utilizes Empirical Wavelet Transform (EWT) to decompose AE signals into mono-components adaptively followed by calculation of the correlated kurtosis (CK) at certain time intervals of these components. By comparing these CK values, the resonant frequency of the rolling bearing can be determined. Then the fault characteristic frequencies are found by spectrum envelope. Both simulation signal and rolling bearing AE signals are used to verify the effectiveness of the proposed method. The results show that the new method performs well in identifying bearing fault frequency under strong background noise.

## 1. Introduction

Rolling bearings are widely used in industry equipment and are key components of rotating machinery. Bearings are inevitably damaged because of overload and long-time operation. Fault bearings make vibration of equipment exceeding operation standard. Badly damaged bearings even lock the whole system and destroy the rotating axle, which initiates downtime and financial loss [1]. Therefore monitoring bearing fault is of great importance to ensure a safe operation of a machine.

Various kinds of monitoring technologies are employed to detect bearing faults, such as encoder, oil, ferro-graphic, vibration and acoustic emission (AE) [2]. Among them, the vibration signal is the most widely used. However, since AE signal can detect early faults of machinery components, the bearing fault monitoring based on AE has been of interest to researchers in recent years [3]. AE is transient stress wave rapidly released by localized energy [4]. During the operation of bearing, main sources of AE include impacting and friction of bearing rollers passing over outer and inner race and fatigue damage of bearing components [5]. Both traditional AE characteristic parameters and newer signal processing methods are employed to analyze AE signals.

Traditional AE characteristic parameters are studied thoroughly to detect rolling bearing faults [6,7,8]. The research results indicate that AE technology has better performance over vibration analysis for monitoring early deterioration of rolling bearing [3]. Morhain and Mba studied the relationship of RMS, energy and count values with rotational speed, load and the size of the faults [8]. Abdullah first used duration time of AE pulses to indicate the defect size of the outer race [9]. Since AE signal is always disturbed by noise, Khamis first proposed the Energy Index (EI) technique to enhance the AE burst buried in random noise. The EI index is utilized to detect incipient faults of bearings [5].

A variety of signal processing methods based on AE technology are used to monitoring bearing condition. Kurtogram and spectral kurtosis are applied to enhance AE events [10]. Kilundu first investigated cyclo-stationarity characteristic of AE bearing signals and used it to find outer race defects [11]. Pandya utilized Hilbert-Huang Transform (HHT) to obtain the time-frequency distribution of AE bearing signals. Then the K-nearest neighbor algorithm based on asymmetric proximity function was employed to classify the signal features, by which the reliability of bearing fault diagnosis increased [12]. Zvokelj proposed a new method, Ensemble Empirical Mode Decomposition-based multiscale Independent Component Analysis, to monitoring bearing fault. The algorithm can not only detect bearing faults but also de-noise AE signals [13]. Farzad Hemmati further developed the bearing fault detection technology by optimizing the ratio of Kurtosis and calculating Shannon entropy [14]. Mourad Kedadouche first used EWT to AE bearing signals to find frequency band which is excited more than other bands. The proposed method successfully detected defects as low as 40 microns on bearing outer race [15]. 

Although a lot of investigations are devoted to detect bearing faults, there are still two problems to be solved. Firstly, most of the methods are only effective in outer race defect, but ineffective in inner race fault due to signal attenuation. Secondly, AE signals are easily disturbed by background noise, so method with good anti-noise performance is needed. Therefore, in this paper a new method based on Empirical Wavelet Transform (EWT) and correlated kurtosis (*CK*) is proposed. Analysis on simulated signal proves that the resonant frequency band and fault characteristic frequency can be effectively obtained with this method. The rolling bearing experiment is carried out and AE signals of defect bearing are used to prove the efficiency of the proposed method.

The rest of the paper is organized as follows. The EWT algorithm and *CK* are introduced in Section 2. In Section 3, the proposed method is applied to simulation signal to validate its effectiveness. Then, in Section 4, rolling bearing experiment is carried out and the method is employed to detect the inner race fault of bearing. Section 5 summarizes the conclusion.

## 2. Introduction to the Theory

### 2.1. Empirical Wavelet Transform

EWT divides signals into frequency sub-bands adaptively according to the information located in the spectrum of the original signal [16,17]. The signal can be decomposed into sub-bands by a set of wavelet construction. The detail of the EWT algorithm is as follows. 

Suppose that the signal has a Fourier support within [0,π]. The spectrum can be divided into Nth contiguous partitions. Each component has the frequency bandwidth as: $\Lambda_{n}=\left[\omega_{n-1},\omega_{n}\right]$, where $\omega_{0}=0$, $\omega_{N}=\pi$, $\cup_{n=1}^{N}\Lambda_{n}=\left[0,\pi \right]$. The empirical scaling function and the empirical wavelets are shown in Equations (1) and (2) respectively. 

(1) $${\overset{\wedge }{\Phi }}_{n}\left(\omega \right)=\left\{\begin{array}{cc} 1 & \mathrm{if}\;\left|\omega \right|\leq \omega_{n}-\tau_{n}\\ \cos\left[\frac{\pi }{2}\beta \left(\frac{1}{2\tau_{n}}\left(\left|\omega \right|-\omega_{n}+\tau_{n}\right)\right)\right] & \mathrm{if}\;\omega_{n}-\tau_{n}\leq \left|\omega \right|\leq \omega_{n}+\tau_{n}\\ 0 & \mathrm{otherwise} \end{array}\right\},$$

(2) $${\overset{\wedge }{\Psi }}_{n}\left(\omega \right)=\left\{\begin{array}{cc} 1 & \mathrm{if}\;\omega_{n}+\tau_{n}\leq \left|\omega \right|\leq \omega_{n-1}-\tau_{n+1}\\ \cos\left[\frac{\pi }{2}\beta \left(\frac{1}{2\tau_{n-1}}\left(\left|\omega \right|-\omega_{n+1}+\tau_{n+1}\right)\right)\right] & \mathrm{if}\;\omega_{n+1}-\tau_{n+1}\leq \left|\omega \right|\leq \omega_{n+1}+\tau_{n+1}\\ \sin\left[\frac{\pi }{2}\beta \left(\frac{1}{2\tau_{n}}\left(\left|\omega \right|-\omega_{n}+\tau_{n}\right)\right)\right] & \mathrm{if}\;\omega_{n}-\tau_{n}\leq \left|\omega \right|\leq \omega_{n}+\tau_{n}\\ 0 & \mathrm{otherwise} \end{array}\right\},$$

Here, $T_{n}$ is the transition phase corresponding to the $\omega_{n}$, which has the width of $2\tau_{n}$. The β(χ) is an arbitrary function. In this paper, it is defined as follows:

(3) $$\beta \left(\chi \right)=\chi^{4}\left(35-84\chi +70\chi^{2}-20\chi^{3}\right),$$

Similar to the continuous wavelet transformation (CWT), the detail coefficients and approximation coefficients can be obtained by the empirical wavelets and scaling function, the functions are shown as follows:

(4) $$W_{\chi }\left(n,t\right)=\int \chi \left(\tau \right)\psi_{n}\left(\tau -t\right)d\tau =F^{-1}\left(\overset{\wedge }{X}\left(\omega \right)\bar{{\overset{\wedge }{\psi }}_{n}\left(\omega \right)}\right),$$

(5) $$W_{\chi }\left(0,t\right)=\int \chi \left(\tau \right)\phi_{1}\left(\tau -t\right)d\tau =F^{-1}\left(\overset{\wedge }{X}\left(\omega \right)\bar{{\overset{\wedge }{\phi }}_{1}\left(\omega \right)}\right),$$

The extracted modes can be obtained as below:

(6) $$f_{0}\left(t\right)=W_{\chi }\left(0,t\right)\times \phi_{1}\left(t\right),$$

(7) $$f_{k}\left(t\right)=W_{\chi }\left(k,t\right)\times \psi_{k}\left(t\right),$$

In order to ensure the decomposition has a tight frame, Equation 8 must be satisfied.

(8) $$\gamma <\min(\frac{\omega_{n+1}-\omega_{n}}{\omega_{n+1}+\omega_{n}}),$$

where $\gamma =\frac{\tau_{n}}{\omega_{n}}$. More detailed information can be found in reference [16].

### 2.2. Correlated Kurtosis

According to reference [18], the definition of *CK* is shown as follows:

(9) $$CK_{M}(T)=\frac{\sum_{n=1}^{N}(\prod_{m=0}^{M}y_{n-mT}{)}^{2}}{{\left(\sum_{n=1}^{N}y_{n}^{2}\right)}^{M+1}},$$

Here, M behalves the order of shift. N is the length of the input signal. T is the sampling point which can be calculated as following:

(10) $$T=\frac{fs}{f_{c}},$$

where fs is the sampling frequency; $f_{c}$ is the fault character frequency. In practice, the T varies in a certain range due to the fluctuation of rotational speed.

From the definition, it is known that *CK* reveals the periodicity of signal. If *T* is the period of fault, the *CK* can indicate the occurrence of fault by its magnitude. The frequency band with maximum value of *CK* is the frequency band which is excited most by the fault characteristic frequency corresponding to *T*.

For rolling bearing, the fault frequencies are always modulated to the resonant frequency and the higher order resonant frequencies. Since the fault frequencies of rolling bearing have obvious periodicity, the *CK* at time interval *T* that equals to the fault period will achieve higher value at resonant frequency bands. Therefore, the frequency bands with the highest *CK* value can be considered as the resonant frequency or the higher order resonant frequencies. 

**Figure 1:**
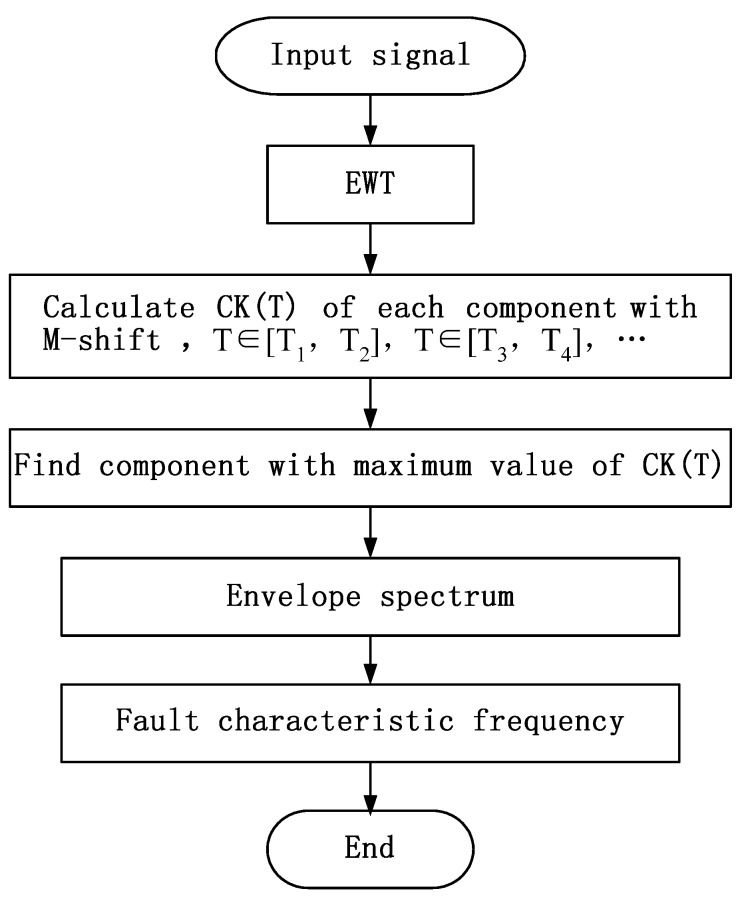
Flowchart of algorithm.

According to the analysis above, a new method is proposed to find resonant frequency and fault frequency by EWT and *CK*. The whole procedure of the proposed algorithm is shown as a flowchart (Figure 1).

## 3. Simulation Verification

To verify the proposed method, a simulation signal is employed. When a local fault in a rolling bearing emerges, harmonic components and periodical impulses will be generated. These frequency components are modulated by resonance frequency of bearing and decay exponentially [19]. 

The simulation signal of defect bearing is expressed as follows:

(11) $$x\left(t\right)=\sum_{k=1}^{M}A_{0}s\left(t-kT\right)+n(t),$$

(12) $$s\left(t\right)=e^{-\zeta t}\cos(2\pi f_{n}t+\varphi_{0}),$$

where $A_{0}$ is the impulse response amplitude; ζ is the damping ratio of the impulse; M is the number of impulses; T is the period of the impulses which is related to the fault characteristic frequency $f_{c}$; $f_{n}$ is the resonance frequency; $\varphi_{0}$ is the primary phase; n(t) is white noise. The value of these parameters are displayed as following:

$$A_{0}=1;\;\zeta =160;\;M=3;\;T=0.05 s;\;f_{c}=20\mathrm{Hz};\;f_{n}=1300\mathrm{Hz};\;\varphi_{0}=0;\;SNR=-20\mathrm{dB}.$$

**Figure 2:**
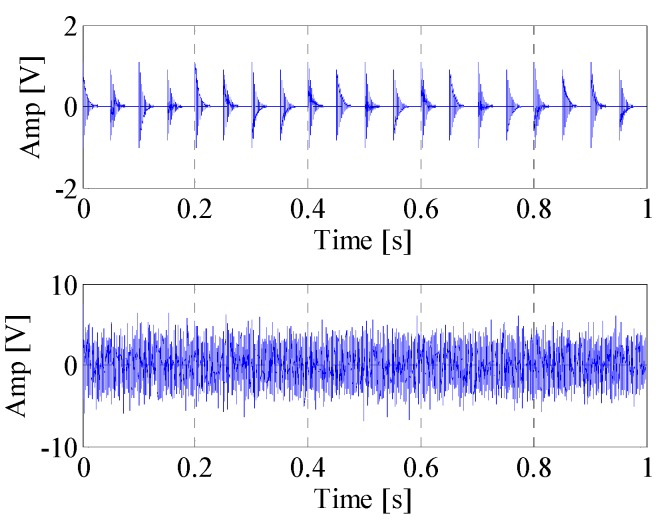
The simulated impulse signal and simulated signal with noise.

**Figure 3:**
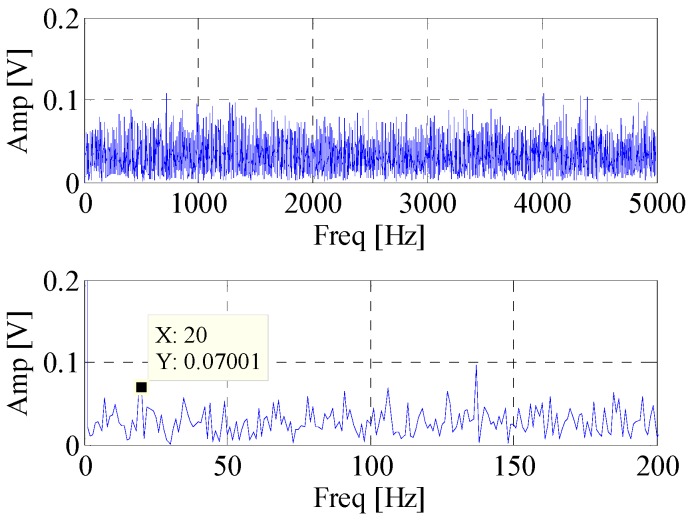
The spectrum and envelope spectrum of simulation signal with noise.

**Figure 4:**
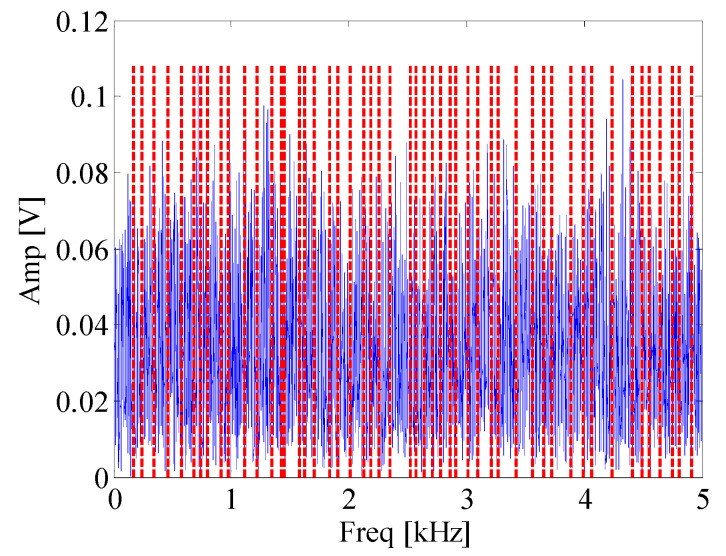
The segmentation of spectrum of simulation signal.

**Figure 5:**
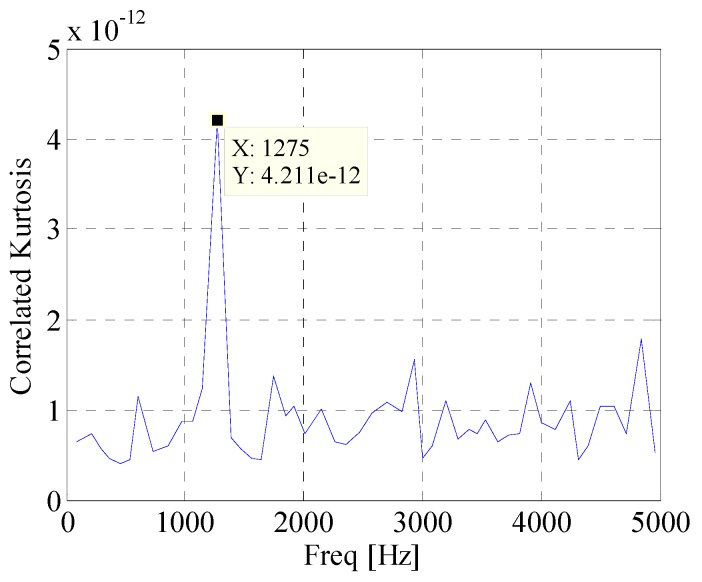
Correlated kurtosis (*CK*) value of components with time interval equals to T.

**Figure 6:**
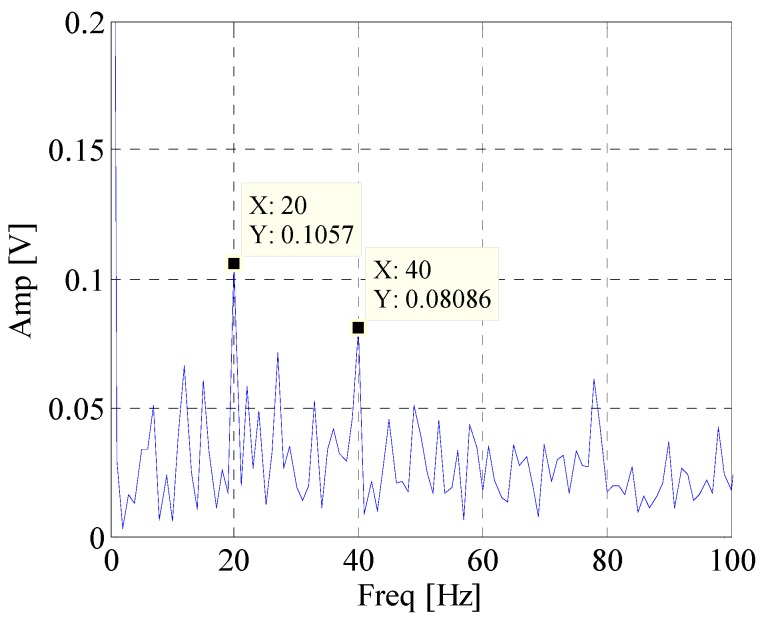
Envelope spectrum of component with maximum *CK* value.

First, the EWT is utilized to decompose the simulated signal adaptively. Then the *CK* of these modes are calculated at the given value of T. The waveforms of the simulated signal with and without noise are shown in Figure 2. Figure 3 displays the spectrum and envelope spectrum of the simulation signal with noise. It shows that the frequency 20 Hz is buried in strong background noise. The spectrum segmentation by EWT is shown in Figure 4. It is hard to find resonant frequency only by the amplitude of the spectrum. Figure 5 is the *CK* value of each component at time interval T. The peak frequency appears at 1275 Hz. It approximates to the true value of resonant frequency, which is 1300 Hz. The error between them can be ascribed to the strong noise. Figure 6 is the envelope spectrum of frequency band centered around 1275 Hz. In Figure 6, the frequency 20 Hz and its harmonics are obvious. It is clearly that the *CK* performs well in identifying resonant frequency and periodic frequency under low SNR.

## 4. Experimental Verification

**Figure 7:**
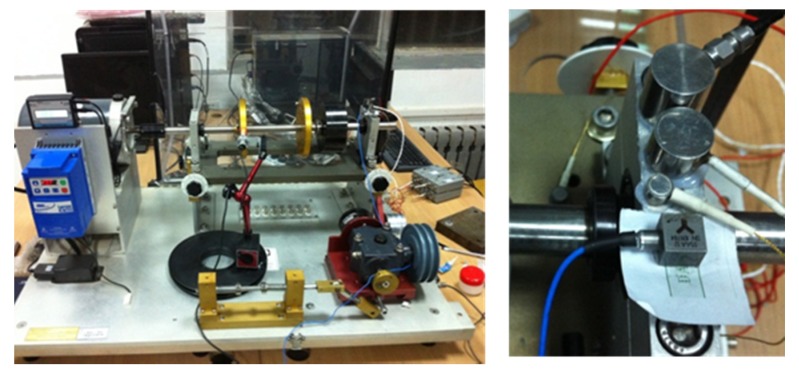
Experimental set up.

Our rolling bearing experiment is explained in this section. The experimental setup is displayed in Figure 7. It consisted of motor diver, shaft, bearings, bearing housing, inertia disc and control system. The shaft was supported by two bearings. The left one was normal and the right one defective. An inner race fault rolling bearings was used in this experiment. The defect was caused artificially. The rotation speed of the experiment was 3000 rpm. 

The AE signals were picked up in the experiment. By comparing performance of three kinds of AE sensors, Nano 30 was selected. The AE sensor was mounted on the top surface of bearing housing (Figure 7). The parameters of Nano 30 is shown in Table 1. The original AE signals were amplified by 40 dB. The AE signal collection system was PCI-II (Physical Acoustics Corporation, Boston, MA, USA). The sample rate was 500 kHz and the AE threshold level was set to 40 dB.

When defects occur in the rolling bearings, some certain frequency components will be generated according to the rotational speed, geometric parameters of bearings and the type of faults [20]. The formulae of the defective frequencies are as follows:

Ball-pass frequency of outer race:

(13) $$\mathrm{BPFO}\;=\;\frac{nf_{r}}{2}\{1-\frac{d}{D}\cos\phi \},$$

Ball-pass frequency of inner race:

(14) $$\mathrm{BPFI}\;=\;\frac{nf_{r}}{2}\{1+\frac{d}{D}\cos\phi \},$$

Fundamental train frequency (cage speed):

(15) $$\mathrm{FTF}\;=\;\frac{f_{r}}{2}\{1-\frac{d}{D}\cos\phi \},$$

Ball (roller) spin frequency:

(16) $$\mathrm{BSF}=\frac{Df_{r}}{2d}\{1-{\left(\frac{d}{D}\cos\phi \right)}^{2}\}$$

Here, *f_r_* is the shaft rotation frequency, *n* is the number of rolling elements, and *a* is the contact angle of the load. *D* and *d* represent the diameter and pitch diameter of rolling element respectively. 

The fault feature frequencies of the rolling bearing are listed in Table 2.

**Figure 8:**
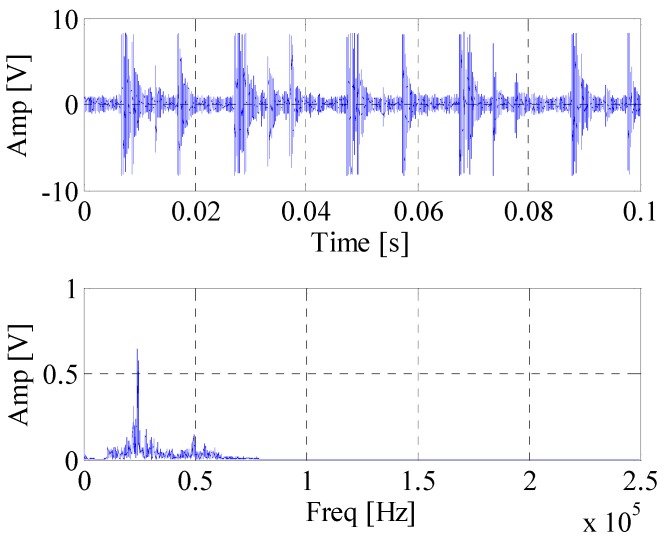
The waveform and spectrum of AE bearing signal.

**Figure 9:**
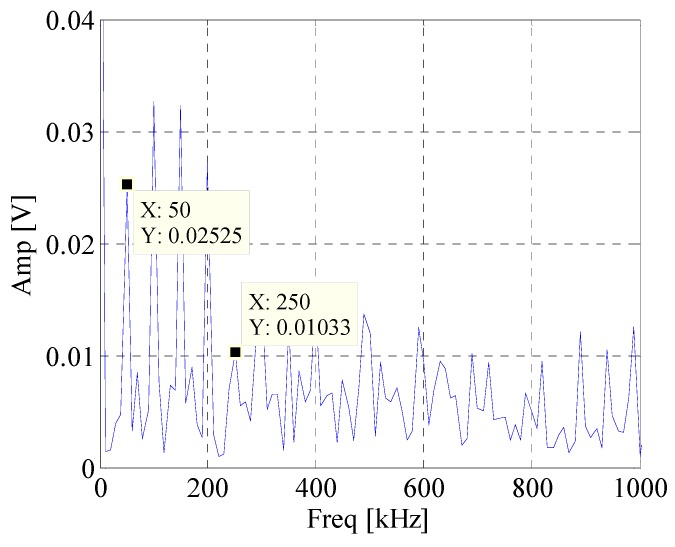
The envelope spectrum of AE bearing signal.

**Figure 10:**
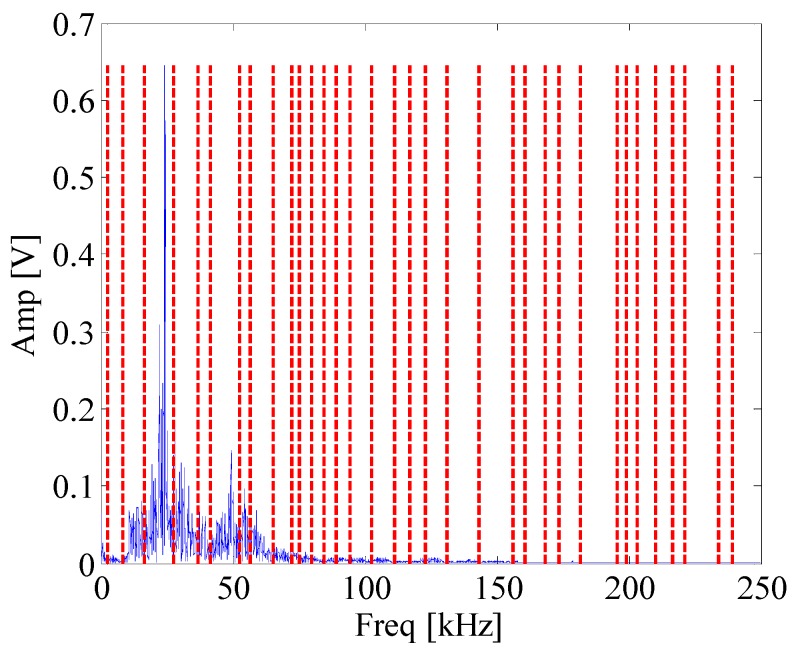
The segmentation of spectrum of bearing signal.

**Figure 11:**
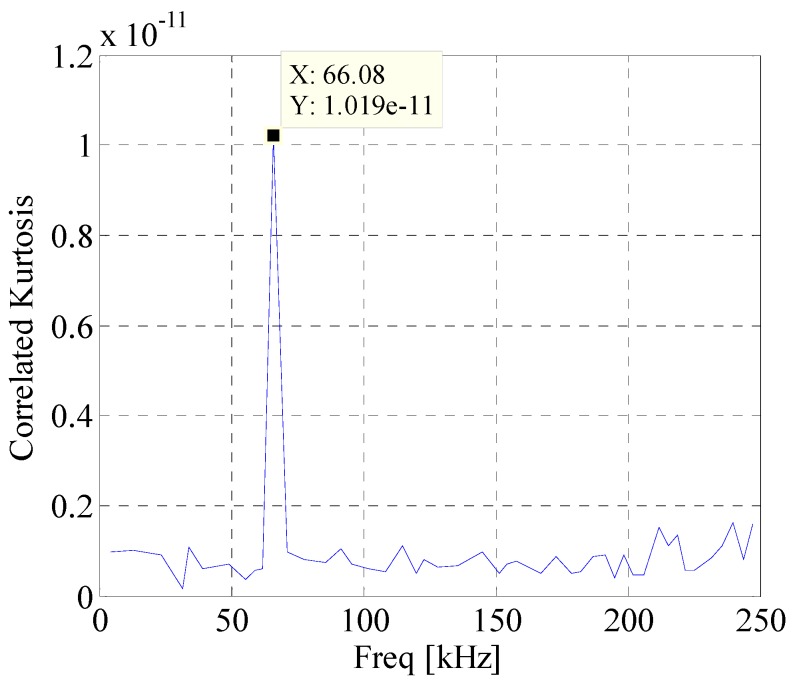
*CK* value of components at time interval *T*.

**Figure 12:**
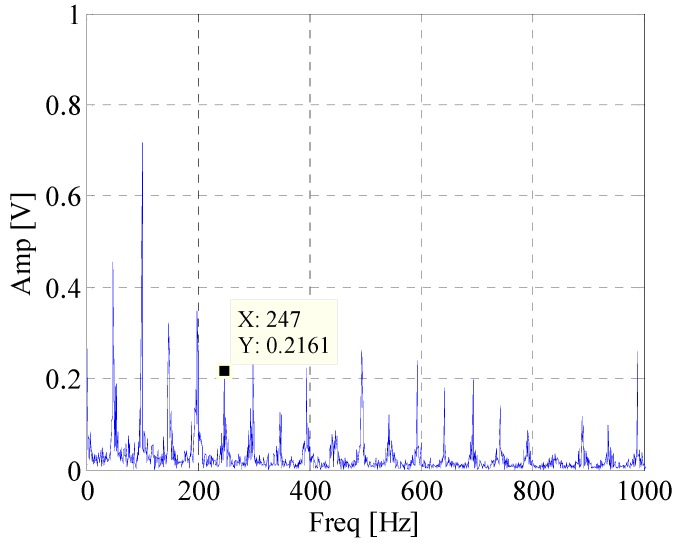
Envelope spectrum of component with maximum *CK* value.

Figure 8 shows waveform and spectrum of rolling bearing with inner race faults. Figure 9 is the envelope spectrum of AE bearing signal. It can be seen that the rotating frequency and its harmonics are obvious, but the BPFI which is defined in Equation (14) and provided in Table 2 cannot be found in the figure. Then the EWT is carried out to decompose the bearing AE signal. The segmentation of the spectrum is shown in Figure 10. Figure 11 displays *CK* value of components at time interval T which equals to inner fault period. The maximum value appears at 66k Hz. It means that this frequency band is excited most by the inner race fault frequency. Figure 12 is the spectrum envelope of this frequency band. It is clear that the rotating frequency is also modulated to this band. However, in Figure 12, the amplitude of the inner race fault frequency increases and the higher order of inner race fault frequencies are clear. The result shows that the proposed method can improve the inner race fault detection of rolling bearing.

It must be pointed out that the time interval T is the reciprocal of fault character frequency and the fault character frequency of rolling bearing is proportional to rotating speed, so the speed variation will affect the time interval T. If the speed variation is small, the accurate time interval T changes within certain range centered around the reciprocal of the fault character frequency. If the speed variation is large, the proposed method will not work.

## 5. Conclusions

In this paper, a new method is proposed to detect defects in rolling bearing. EWT is utilized to decompose AE signals into different components. *CK* of these components at a certain time interval T are calculated. Components with the largest *CK* value is considered to be the resonant frequency. Then envelope demodulation is carried out to obtain the fault feature characteristics of corresponding frequency bands. Simulated and experimental signals were employed to verify the effectiveness of the method. Two conclusions can be drawn as follows:

1. With the right time interval T, the resonant frequency of the rolling bearing system can be obtained by calculating the *CK* value.
2. Weak fault features can be identified by the proposed method under low SNR conditions.

Future research work will be devoted to enhance the fault frequencies modulated by different resonant frequencies and estimation of the accurate time interval T under slight speed fluctuation will also be carried out.

## Figures and Tables

**Table 1:** Parameters of Nano30.

Parameters	Range
Operating Frequency Range	125–750 KHz
Resonant Frequency	300 KHz
Temperature Range	−65–177 °C

**Table 2:** Fault character frequency of rolling bearing.

Rotation Speed/rpm	BPFO/Hz	BPFI/Hz	BSF/Hz
3000	152.4	247.5	99.6
